# A randomised, phase II study of intetumumab, an anti-*α*_v_-integrin mAb, alone and with dacarbazine in stage IV melanoma

**DOI:** 10.1038/bjc.2011.183

**Published:** 2011-07-12

**Authors:** S O'Day, A Pavlick, C Loquai, D Lawson, R Gutzmer, J Richards, D Schadendorf, J A Thompson, R Gonzalez, U Trefzer, P Mohr, C Ottensmeier, D Chao, B Zhong, C J de Boer, C Uhlar, D Marshall, M E Gore, Z Lang, W Hait, P Ho, on the behalf of the CNTO 95 Investigators

**Affiliations:** 1 The Angeles Clinic and Research Institute, 2001 Santa Monica Boulevard, Suite 560W, Santa Monica, CA, USA; 2 New York University, New York, NY, USA; 3 Universitätsklinikum Essen, Essen, Germany; 4 University Medical Center, Mainz, Germany; 5 Emory University, Atlanta, GA, USA; 6 Klinik und Poliklinik für Dermatologie, Venerologie und Allergologie, Medizinische Hochschule Hannover, Hannover, Germany; 7 Oncology Specialists, S.C., Park Ridge, IL, USA; 8 Universitätsklinikum Mannheim, Mannheim, Germany; 9 Seattle Cancer Care Alliance, Seattle, WA, USA; 10 University of Colorado HSC, Aurora, CO, USA; 11 Charité Universitätsmedizin Berlin, Berlin, Germany; 12 Elbeklinikum Buxtehude, Buxtehude, Germany; 13 Cancer Research UK Clinical Centre, Southampton, UK; 14 Royal Free Hospital, London, UK; 15 Centocor Oncology Research and Development, Inc., Malvern, PA, USA; 16 Centocor B.V., Leiden, The Netherlands; 17 Royal Marsden Hospital, London, UK

**Keywords:** intetumumab, melanoma, *α*_v_ integrins, dacarbazine, CNTO 95

## Abstract

**Background:**

*α*_v_ integrins are involved in angiogenesis and melanoma tumourigenesis. Intetumumab (CNTO 95) is a fully human anti-*α*_v_-integrin monoclonal antibody.

**Methods:**

In a multicentre, randomised, phase II study, stage IV melanoma patients were randomised 1 : 1 : 1 : 1 to 1000 mg m^−2^ dacarbazine+placebo (*n*=32), 1000 mg m^−2^ dacarbazine+10 mg kg^−1^ intetumumab (*n*=32), 10 mg kg^−1^ intetumumab (*n*=33), or 5 mg kg^−1^ intetumumab (*n*=32) q3w. The primary endpoint was progression-free survival (PFS). Secondary endpoints included overall survival (OS), objective response rate (ORR), adverse events, and pharmacokinetics.

**Results:**

No statistically significant differences in efficacy were observed between groups. In the dacarbazine+placebo, dacarbazine+intetumumab, 10 mg kg^−1^ intetumumab, and 5 mg kg^−1^ intetumumab groups, median PFS was 1.8, 2.5, 1.4, and 1.4 months; median OS was 8, 11, 15, and 9.8 months; and ORR of complete+partial response was 10, 3, 6, and 0%. Nonlinear intetumumab pharmacokinetics and potential intetumumab–dacarbazine interactions were observed. Transient, asymptomatic, nonrecurring, grade 1–2, uveitic reactions that resolved spontaneously or with topical steroids were seen in 22–30% of intetumumab-treated patients. Low-grade infusion-reaction symptoms (headache, fatigue, nausea, vomiting, fever, chills) were observed, as expected, in 16–73% of dacarbazine-treated patients. No intetumumab-related myelosuppression, laboratory/electrocardiogram abnormalities, or deaths occurred.

**Conclusion:**

With its favourable safety profile and a nonsignificant trend towards improved OS, intetumumab merits further investigation in advanced melanoma.

Melanoma is the eighth most prevalent cancer in the United States (Wingo *et al*, 1995), with a lifetime incidence of 1 in 55 males and 1 in 82 females (Jemal *et al*, 2004). The worldwide incidence of melanoma is increasing, especially in Caucasian populations; estimates suggest a doubling every 10–20 years (Lens and Dawes, 2004). Patients with stage IV melanoma have a poor overall prognosis as reflected by median survival ranging from 6 to 10 months (Sirott *et al*, 1993; Stadelmann *et al*, 1997; Chapman *et al*, 1999).

Dacarbazine has been the standard treatment for stage IV melanoma for the past 30 years, despite incomplete and short-lived responses without improvement in survival (Hill *et al*, 1984; Chapman *et al*, 1999). High-dose interleukin-2 is approved for the treatment of melanoma in the US, but, unlike dacarbazine, is associated with significant toxicity (Jonasch and Haluska, 2001; Atkins, 2002). Numerous studies comparing dacarbazine with a new agent alone or in combination have failed to demonstrate significant impact on survival (Chapman *et al*, 1999; Middleton *et al*, 2000; Eggermont and Kirkwood, 2004; Bedikian *et al*, 2006; McDermott *et al*, 2008). Therefore, more effective treatments are needed.

Integrins are essential to the processes of tumour growth and metastasis. They are a diverse family of transmembrane receptor proteins that facilitate cellular survival and differentiation. The *α*_v_ integrin subfamily consists of at least five members, including *α*_v_*β*_1_, *α*_v_*β*_3_, *α*_v_*β*_5_, *α*_v_*β*_6_, and *α*_v_*β*_8_. *α*_v_ integrins are involved in angiogenesis and are widely overexpressed in numerous human cancers including melanoma (Tucker, 2003). Integrin *α*_v_*β*_3_ is preferentially expressed on vertical growth phase primary melanoma and metastatic lesions but is not detectable on melanocytes, nevi, or radial growth phase primary melanoma (Albelda *et al*, 1990; Van Belle *et al*, 1999). Overexpression of *α*_v_ integrins activates and modulates signalling pathways that enable tumour cells to switch to an invasive phenotype (Seftor *et al*, 1999). Preclinical studies using forced expression of *α*_v_ integrins, integrin-antagonist antibodies, and Arg-Gly-Asp inhibitors of integrins have demonstrated that *α*_v_*β*_3_ plays a critical role in melanoma cell proliferation, survival, metastasis, and disease progression (Felding-Habermann *et al*, 1992; Petitclerc *et al*, 1999; Trikha *et al*, 2004; Mulgrew *et al*, 2006).

The importance of angiogenesis in the growth and metastasis of solid tumours, including melanoma, is well documented. In melanoma, correlations between increasing vascularity within the primary tumour and the incidence of distant metastasis have also been reported (Srivastava *et al*, 1988). Integrins *α*_v_*β*_3_ and *α*_v_*β*_5_ play important roles in angiogenesis, and antibodies directed against *α*_v_*β*_3_ disrupt intratumoural neovascularisation and induce regression of tumour vascularisation in melanoma models (Mahabeleshwar and Byzova, 2007).

Intetumumab (formerly named CNTO 95) is a fully human monoclonal antibody that recognises all members of the *α*_v_ integrin family and has anti-angiogenic and antitumour properties. This pan anti-*α*_v_ integrin antibody binds *α*_v_ integrins with high affinity and specificity, resulting in inhibition of cell adhesion, migration, proliferation, and invasion of both tumour and endothelial cells *in vitro* (Trikha *et al*, 2004). *In vivo* growth of human melanoma tumours was also significantly reduced by intetumumab binding to *α*_v_ integrins (Trikha *et al*, 2004). In phase I studies, intetumumab was well tolerated at doses ranging from 3 to 10 mg kg^−1^ (O’Day *et al*, 2006). Clinical activity in phase I included a partial response (PR) in a patient with angiosarcoma, a tumour of malignant endothelial cells (Mullamitha *et al*, 2007). Here, we report findings from a multicentre, randomised, phase II study designed to assess the efficacy and safety of intetumumab, alone and in combination with dacarbazine, as compared with dacarbazine monotherapy in patients with stage IV melanoma.

## Patients and methods

### Patients

Patients ⩾18 years of age were required to have histologically confirmed stage IV melanoma according to the American Joint Committee on Cancer criteria (Balch *et al*, 2001, 2002), radiographically measurable disease (as defined by Response Evaluation Criteria in Solid Tumours (RECIST)) or measurable skin lesions, no clinical or radiological evidence of central nervous system metastases, Eastern Cooperative Oncology Group (ECOG) performance status (ECOG, 2006) of ⩽2, and life expectancy ⩾3 months. Patients were excluded for any previous use of chemotherapy for melanoma and any previous radiotherapy to target lesions. The use of investigational drugs, systemic cancer therapy, or generalised radiotherapy was not permitted within 1 month of first administration of the study agent. Concurrent immunotherapy, biotherapy, radiotherapy, chemotherapy, or investigational therapy was prohibited. The institutional review board or ethics committee for each study site approved the protocol. All patients provided written informed consent.

### Study design and treatment

In this study, patients across 30 centres in the United States, United Kingdom, and Germany were randomly assigned 1 : 1 : 1 : 1 to receive 1000 mg m^−2^ dacarbazine+placebo (active control arm), 1000 mg m^−2^ dacarbazine+10 mg kg^−1^ intetumumab, 10 mg kg^−1^ intetumumab monotherapy, or 5 mg kg^−1^ intetumumab monotherapy. Randomisation was stratified by site of metastases and ECOG performance status at baseline. Patients received each assigned study agent once every 3 weeks for up to 8 cycles. Patients who responded to treatment with stable disease (SD) or better could receive extended dosing. All patients were followed for survival for up to 2 years. Commercially available dacarbazine was administered intravenously over 60 (±30) minutes. Intetumumab or placebo (saline) was administered over 2 h (±15 min) after dacarbazine administration.

Patients in the blinded dacarbazine-containing arms who could not tolerate dacarbazine were allowed to cross over to open-label 10 mg kg^−1^ intetumumab monotherapy, and those on dacarbazine monotherapy who experienced progressive disease (PD) were allowed to cross over to open-label dacarbazine+10 mg kg^−1^ intetumumab. Intetumumab monotherapy arms were open-label. Blinding was maintained until symptomatic deterioration, study-agent crossover, or serious unexpected adverse reaction.

### Study assessments

The primary endpoint was progression-free survival (PFS), defined as time from randomisation to the earliest date of documented PD, documented symptomatic deterioration, or death. Major secondary endpoints included overall response rate (tumour response: complete response (CR) and PR, CR) and overall survival (OS; time from randomisation to date of death or last contact). Patients were followed for survival every 3 months until death, loss to follow-up, withdrawal of consent, or the end of the planned 2-year follow-up.

Safety assessments included the monitoring of adverse events (AEs), serious adverse events (SAEs), allergic/hypersensitivity reactions, ophthalmic events, infusion reactions, and significant laboratory or electrocardiogram results throughout the study. Adverse events and SAEs were monitored through the planned follow-up at 3 and 6 months after the last study visit.

Blood samples were obtained at cycle 1 pre-infusion and cycle 3 pre- and post-infusion for a limited pharmacokinetic assessment. Serum intetumumab concentrations were measured using a validated immunoassay with a lower limit of detection of 0.2 *μ*g ml^−1^.

Tumours were assessed clinically or radiologically with computed tomography or magnetic resonance imaging within 28 days of first study-agent infusion; within 1 week of the end of cycles 2, 4, 6 and 8; and at the planned follow-up at 3 and 6 months after the last study visit. Objective tumour response was evaluated for target and non-target lesions using modified RECIST criteria (Therasse *et al*, 2000) by a site radiologist blinded to treatment assignment. Four weeks after documented CR or PR, additional radiologic and clinical assessments were performed to confirm the response.

Optional tumour biopsies were obtained from consenting patients before and after intetumumab treatment to evaluate changes in the expression level of *α*_v_ integrin and intetumumab binding by immunohistochemistry.

### Statistical analysis

The sample size was not determined based on statistical power consideration but was chosen to provide preliminary data on safety and efficacy. The primary endpoint was analysed based on the intention-to-treat population. PFS was censored at the date of the last adequate assessment for PD. Survival probabilities were estimated using the Kaplan–Meier method for each treatment arm. The 95% confidence intervals around the Kaplan–Meier estimates were constructed via Greenwood's formula (Collett, 1994). Treatment comparison with dacarbazine+placebo was performed using the stratified Log-rank test and Cox's regression (Cox, 1972), with site of metastases (M1a/b *vs* M1c) and ECOG performance status (0 and 1 *vs* 2) at baseline as stratification factors. Although there was no prespecified criterion for improvement in PFS (compared with the dacarbazine+placebo arm) and the determination of treatment differences was focused on discerning trends, *P*-values were calculated from the Log-rank test and hazard ratios with 95% confidence intervals were calculated from Cox's regression, stratified by site of metastases and ECOG performance status at baseline.

The following subgroup analyses were prespecified to compare each treatment arm with dacarbazine+placebo for PFS and OS: age (<65 *vs* ⩾65), gender (male *vs* female), baseline lactate dehydrogenase (LDH; >normal *vs* ⩽normal), site of metastases (M1a/b *vs* M1c), geographic region (Europe *vs* North America), and uveitis/iritis (presence *vs* absence).

Descriptive statistics were used to summarise the secondary endpoints and the exploratory analyses. Overall survival duration was censored at the date of last contact for remaining patients. Treatment arm comparisons were made using the parameter estimate and 95% confidence interval. Although no hypothesis testing was performed, nominal *P*-values were presented as a measure of the strength of associations.

Safety analyses were based on actual treatment received.

## Results

### Patients and treatment

A total of 129 patients were randomised. Among these patients, 20 (16%) did not meet the protocol-defined entry criteria owing to inadequate bone marrow, liver, and renal function laboratory criteria (*n*=12, 9%); medical history criteria deviation (*n*=5, 4%: 4 patients had concomitant/previous malignancy and 1 patient had a history of uveitis); metastatic melanoma criteria deviation (*n*=2, 2%: 1 patient had stage III melanoma and 1 patient had central nervous system metastases); and concurrent immuno/bio/radio/chemotherapy or investigative therapy criteria deviation (*n*=2, 2%).

**Figure 1:**
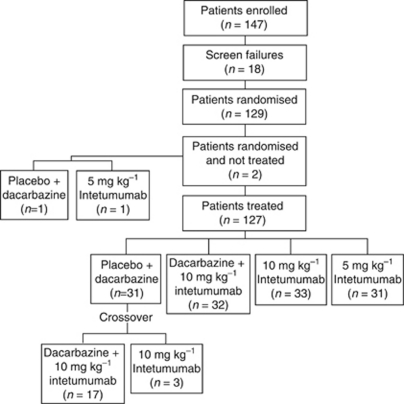
Patient flow. Twenty (15.5%) randomised patients did not meet the protocol-defined entry criteria, the majority (*n*=12, 9.3%) because of laboratory criteria violations. The most significant protocol violation was reported for one patient in the 10 mg kg^−1^ intetumumab+dacarbazine arm who had stage III melanoma at baseline and was excluded from analysis.

Of the 129 randomised patients, 127 were treated (Figure 1). Baseline patient characteristics are shown in Table 1. Fifty-seven patients (47%) had previous systemic therapy, and 38 (31%) had previous radiotherapy. More patients in the 10 mg kg^−1^ intetumumab-containing arms than in the dacarbazine alone or 5-mg kg^−1^ intetumumab arms had previous systemic therapy. Prognostic characteristics were generally similar among all arms with respect to the proportion of patients who were M1a/b *vs* M1c and had an ECOG score of 0/1 *vs* 2, although a slightly lower proportion of patients in the 10-mg kg^−1^ intetumumab arm had above normal LDH.

With the exception of the dacarbazine+intetumumab arm, which received a median of 3 (range 1–17) treatment cycles, all other arms received 2 median (range 1–20) cycles. Among patients assigned to dacarbazine alone, 17 crossed over to dacarbazine+10 mg kg^−1^ intetumumab and 3 patients crossed over to 10-mg kg^−1^ intetumumab monotherapy, with a median of 2 post-crossover cycles administered in both.

### Safety

A summary of AEs is shown in Table 2. Almost all patients experienced AEs, with those in the dacarbazine-containing arms more frequently experiencing grade 3–4 events compared with those receiving intetumumab only. As expected, haematologic toxicity consisting of myelosuppression was observed exclusively in patients receiving dacarbazine with the exception of one patient with grade 1 anaemia in the 5-mg kg^−1^ intetumumab arm. Interestingly, hypotension, which is associated with dacarbazine infusions, occurred less frequently when intetumumab was administered in combination. The most common AEs observed in patients receiving intetumumab included events commonly associated with infusion reactions such as headache (38–73%), fatigue (23–42%), nausea (23–47%), vomiting (19–39%), fever (19–39%), chills (16–27%), infusion-site pain (0–19%), and extremity pain (0–16%). These events were typically low grade, rarely observed in more than one patient, with the exception of grade 3 headache that never progressed to grade 4. These same AEs were seen with similar frequencies in patients receiving dacarbazine alone.

Two additional AEs, uveitic reactions and diarrhoea, appeared to be associated with intetumumab administration. Twenty-four patients treated with intetumumab and one treated with dacarbazine alone experienced transient, grade 1 or 2, anterior chamber uveitic reactions that differ from classical uveitis by the absence of flare, redness, pain, or visual disturbances. Besides being asymptomatic, the uveitic reactions resolved spontaneously or with topical steroids before the next infusion, did not recur upon subsequent infusions or result in long-term sequelae. Diarrhoea that was usually grade 1 also appeared to occur more frequently (10–22%) with intetumumab than with dacarbazine infusion (10%).

No markedly abnormal laboratory results were observed. Among 70 patients who were monitored for 12-lead electrocardiogram at the final visit, none had clinically significant abnormalities. No treatment-related deaths occurred during the study.

### Pharmacokinetics

Consistent with the experience from phase I studies (O’Day *et al*, 2006), intetumumab pharmacokinetics were nonlinear, with greater than dose-proportional trough serum concentrations at 10 mg kg^−1^ compared with 5 mg kg^−1^. Following administration of 10 and 5 mg kg^−1^ intetumumab alone, the median intetumumab trough serum concentrations were 21.4 and 1.03 *μ*g ml^−1^, respectively, before the cycle 3 infusion compared with peak serum concentrations of 189 and 147 *μ*g ml^−1^, respectively, after the cycle 3 infusion. The addition of dacarbazine to intetumumab appeared to reduce exposure of the latter, as reflected by a median trough serum concentration of 10.9 *μ*g ml^−1^ for intetumumab.

### Efficacy

**Figure 2:**
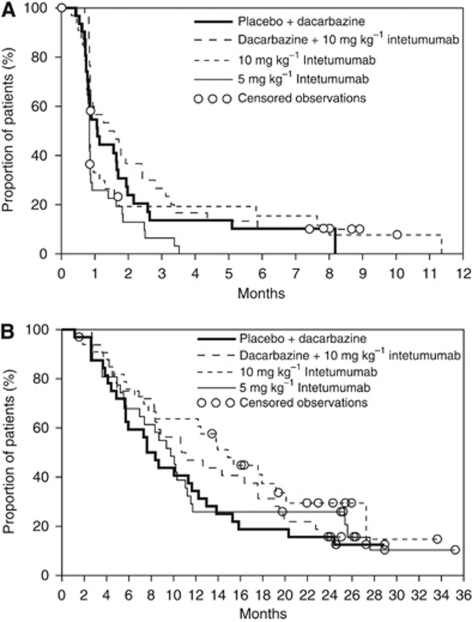
(**A**) Progression-free survival using Kaplan–Meier estimates, randomised patients. (**B**) Overall survival using Kaplan–Meier estimates, randomised patients.

No statistically significant differences were observed in the primary endpoint of PFS across the treatment arms of this study (Table 3, Figure 2A).

Objective tumour responses were uncommon. Six (5%) patients had a PR: 3 in the dacarbazine alone arm, 1 in the 10 mg kg^−1^ intetumumab+dacarbazine arm, and 2 in the 10 mg kg ^−1^ intetumumab monotherapy arm (Table 3). The duration of these responses were 3.9, 7.3, and 10.3+ months in the dacarbazine+placebo arm; 7.0 months in the dacarbazine+intetumumab arm; and 6.3 and 8.2+ months in the 10 mg kg^−1^ intetumumab arm. An additional patient treated with dacarbazine+intetumumab was confirmed to have a late evolving CR, which was considered SD for this analysis. Forty-two (34%) patients had a best overall response of SD. A trend towards increased SD rate was observed with dacarbazine+10 mg kg^−1^ intetumumab (*n*=16, 53%) compared with dacarbazine alone (*n*=10, 32%).

A trend for improved OS was observed in both 10 mg kg^−1^ intetumumab-containing arms compared with the dacarbazine alone arm through the planned 2-year follow-up. Patients receiving 10 mg kg^−1^ in combination with dacarbazine had a median OS of 11 months (HR 0.78 (95% CI 0.45, 1.33)) and those receiving 10 mg kg^−1^ alone had a median OS of 15 months (HR 0.61 (95% CI 0.35, 1.07)) compared with the control arm of dacarbazine alone (8 months) (Table 3). Although not statistically significant, OS in the two 10 mg kg^−1^ intetumumab-containing arms appeared generally more favourable compared with the dacarbazine control arm by Kaplan–Meier analysis (Figure 2B). Notably, the estimated 1-year OS rate for patients receiving 10 mg kg^−1^ intetumumab monotherapy was almost double that for patients receiving dacarbazine alone (64 *vs* 34% Table 3) and nearly 50% greater for patients receiving dacarbazine+intetumumab (47 *vs* 34% Table 3).

Protocol-specified subgroup analyses revealed that the point estimates for improved hazard ratios for OS in the 10 mg kg^−1^ intetumumab-containing arms compared with dacarbazine alone were consistently maintained across the prognostic subgroups of gender (male *vs* female), baseline LDH (>normal *vs* ⩽normal), and site of metastases (M1a/b *vs* M1c) (data not shown).

### Integrin expression

An exploratory analysis of the baseline expression of *α*_v_ integrin in tissue biopsies (*n*=36) demonstrated a trend of SD in patients with higher baseline *α*_v_ expression and of PD in patients with lower *α*_v_ expression. There were no specific correlations with clinical response, OS, or PFS. Changes in *α*_v_ integrin expression in the pre- and post-treatment tissue biopsies (*n*=4) were not significantly associated with OS or PFS based on Cox regression analysis, presumably due to the limited number of paired samples analysed.

## Discussion

This randomised, phase II study evaluated the efficacy and safety of a pan-anti-*α*_v_ integrin monoclonal antibody, intetumumab, alone and in combination with dacarbazine compared with dacarbazine alone in patients with stage IV melanoma. No statistically or clinically meaningful improvements in the primary endpoint of PFS were observed in any of the intetumumab-containing arms. Nevertheless, the trends towards improvement in OS in the two 10 mg kg^−1^ intetumumab-containing arms (11 and 15 months) compared with dacarbazine alone as the active control arm (8 months) are encouraging. Although the lack of correlation between an observed advantage for OS and for PFS appears surprising, an analogous finding of improvement in OS but not PFS in melanoma was recently reported in a phase III study with ipilimumab (Hodi *et al*, 2010). In melanoma, the poor correlation between improvements in PFS and OS in clinical trials likely results from small PFS differences that are not clinically meaningful and from relatively early central nervous system metastases in this disease compared with other solid tumours where it is a later event. Furthermore, this OS improvement for ipilimumab monotherapy did not appear to correlate with its objective response rate of 5% observed in a randomised, phase II study in chemotherapy-naive melanoma patients (Hersh *et al*, 2011). Similarly, the phase III trial of sipuleucel-T (Provenge Dendreon Corp., Seattle, WA, USA) revealed a statistically significant difference in OS but not for time to progression in patients with advanced prostate cancer (Higano *et al*, 2009).

**Figure 3:**
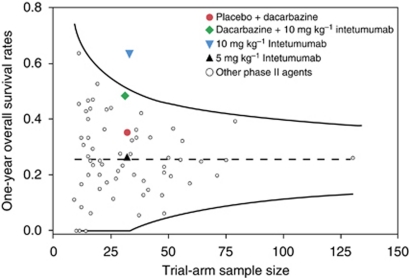
Scatter plot of 1-year overall survival rates from this study and of historical data from the meta-analysis by Korn *et al* (2008). The dotted line at *y*=0.25 represents the mean 1-year overall survival rate and the curved lines represent the 95% confidence bounds of all agents included in the meta-analysis. Reproduced with the kind permission of the American Society of Clinical Oncology from Korn *et al* (2008).

Overall survival has recently been recommended as the optimal primary endpoint in phase II randomised trials in a meta-analysis by Korn *et al* (2008). The authors suggest targeting a 15% improvement over the expected 1-year survival rate of a historical control as a criterion to warrant the further development of a new agent for the treatment of metastatic melanoma. In our study, the estimated 1-year OS rate for patients receiving 10-mg kg^−1^ intetumumab alone was double that for patients receiving dacarbazine alone (64 *vs* 34%) despite allowing crossover to intetumumab in patients who progressed on or were intolerant of dacarbazine. Notably our observed 1-year OS estimate lies above the upper bound of the 95% confidence interval of all OS rates reported in Korn *et al*'s meta-analysis (Figure 3) (Korn *et al*, 2008), suggesting that intetumumab may be efficacious as a single agent for the treatment of metastatic melanoma. Nonetheless, these efficacy results should be interpreted with caution in light of the small sample size and the high crossover rate to intetumumab in the control cohort.

The nonlinear pharmacokinetics of intetumumab and drug–drug interactions between dacarbazine and intetumumab may have contributed to the median OS being longest in the 10 mg kg^−1^ intetumumab arm. Median trough serum concentrations obtained before cycle 3 were nearly 20-fold lower in the 5 mg kg^−1^ than the 10 mg kg^−1^ intetumumab monotherapy arms. Similarly, the dacarbazine+intetumumab combination arm resulted in lower intetumumab exposure as reflected in lower median serum trough concentrations and a 2-fold shorter pharmacokinetic half-life (mean 2.4 days *vs* 5.2 days) for the combination *vs* intetumumab alone arms observed in phase I (data on file). The association between higher expression of *α*_v_ integrin, a critical target of intetumumab, in tumour tissue and SD as opposed to PD is intriguing, although similar correlations were not observed with clinical response, PFS, or OS. Although this does not establish these clinical results as related to intetumumab's anti-integrin activity, a generalised immune stimulation from intetumumab has not been observed in preclinical investigations or in other clinical trials.

The addition of intetumumab to dacarbazine did not appear to significantly increase toxicity compared with dacarbazine alone. Patients in the two intetumumab monotherapy arms did not experience myelosuppression as was observed in the dacarbazine-containing arms. Adverse drug reactions (hypertension, proteinuria, thromboembolism, impaired wound healing) seen with other anti-angiogenic agents such as bevacizumab were not observed in intetumumab-treated patients. However, infusion reactions and uveitic reactions were observed almost exclusively in intetumumab-treated patients and were self-limited and well managed. Infusion reactions occurred despite premedication with antipyretics; other premedication such as anti-emetics should be considered for intetumumab monotherapy in future studies. Twenty-two to 30% of patients in the dacarbazine+10 mg kg^−1^ intetumumab, the 10 mg kg^−1^ intetumumab alone, and the 5 mg kg^−1^ intetumumab alone arms experienced transient, asymptomatic uveitic reactions that typically resolved before the next infusion without recurrence or long-term sequelae. The exact mechanism of the uveitic reactions that differ from classical uveitis is unknown. Based on similar observations in previous animal toxicology and phase I studies of intetumumab, continued monitoring is warranted in future studies.

In conclusion, this randomised, controlled trial demonstrates that treatment with intetumumab at 10 mg kg^−1^ every 3 weeks alone or in combination with dacarbazine was associated with a nonsignificant improvement in OS without corresponding improvement in response rates or PFS. Consistent with the phase I experience, intetumumab displayed a manageable safety profile with infusion reactions and asymptomatic, reversible uveitic reactions as the most clinically relevant AEs. These results further support the clinical evaluation of intetumumab for the treatment of metastatic melanoma in larger clinical studies with the primary endpoint of OS.

## Figures and Tables

**Table 1:** Baseline patient characteristics

	**Dacarbazine+ placebo**	**Dacarbazine+ 10 mg kg^−1^ intetumumab**	**10 mg kg^−1^ Intetumumab**	**5 mg kg^−1^ Intetumumab**
Patients randomised	32	32	33	32
Male	18 (56)	18 (56)	26 (79)	24 (75)
Age (years)	64 (56, 74)	60 (52, 66)	61 (54, 71)	69 (60, 74)
				
*Geographic region*				
Europe	15 (47)	18 (56)	14 (42)	21 (66)
North America	17 (53)	14 (44)	19 (58)	11 (34)
				
*Previous therapy*				
*N*	30	30	32	30
Systemic therapy	11 (37)	18 (60)	17 (53)	11 (37)
Interferon	4 (13)	9 (30)	9 (28)	6 (20)
Radiotherapy	10 (33)	9 (30)	12 (38)	7 (23)
				
*Site of metastasis*				
M1a	2 (6)	4 (13)	4 (12)	6 (19)
M1b	8 (25)	9 (28)	9 (27)	7 (22)
M1c	22 (69)	18 (56)	20 (61)	19 (59)
				
*Eastern Cooperative Oncology Group performance status*				
0	24 (75)	20 (63)	18 (55)	20 (63)
1	7 (22)	11 (34)	14 (42)	11 (34)
2	1 (3)	1 (3)	1 (3)	1 (3)
>Normal lactic dehydrogenase	13 (41)	13 (41)	11 (33)	13 (41)

Data presented as *n* (%) or median (interquartile range).

**Table 2:** Safety

	**Dacarbazine+placebo**			**Dacarbazine+10 mg kg^−1^ intetumumab**			**10 mg kg^−1^ Intetumumab**			**5 mg kg^−1^ Intetumumab**		
	**Total**	**Grade 3**	**Grade 4**	**Total**	**Grade 3**	**Grade 4**	**Total**	**Grade 3**	**Grade 4**	**Total**	**Grade 3**	**Grade 4**
Patients treated^a^	31			32			33			31		
Any adverse event	31 (100)	8 (26)	6 (19)	32 (100)	12 (38)	4 (13)	32 (97)	9 (27)	0	31 (100)	4 (13)	0
												
*General disorders*												
Fatigue	13 (42)	1 (3)	0	10 (31)	0	0	14 (42)	1 (3)	0	7 (23)	0	0
Infusion-site pain	2 (6)	0	0	6 (19)	0	0	0	0	0	0	0	0
Pyrexia	5 (16)	0	0	6 (19)	0	0	13 (39)	1 (3)	0	9 (29)	0	0
Chills	5 (16)	1 (3)	0	5 (16)	0	0	9 (27)	0	0	5 (16)	0	0
Influenza-like illness	2 (6)	1 (3)	0	2 (6)	0	0	3 (9)	0	0	5 (16)	0	0
Pain	1 (3)	0	0	1 (3)	0	0	1 (3)	0	0	5 (16)	0	0
												
*Gastrointestinal disorders*												
Nausea	16 (52)	0	0	15 (47)	0	0	12 (36)	0	0	7 (23)	1 (3)	0
Vomiting	4 (13)	0	0	8 (25)	0	0	13 (39)	1 (3)	0	6 (19)	1 (3)	0
Constipation	6 (19)	0	0	7 (22)	0	0	3 (9)	0	0	3 (10)	0	0
Diarrhoea	3 (10)	0	0	7 (22)	1 (3)	0	6 (18)	0	0	3 (10)	0	0
Abdominal pain	4 (13)	0	0	2 (6)	0	0	1 (3)	1 (3)	0	1 (3)	0	0
												
*Blood and lymphatic disorders*												
Anaemia	6 (19)	2 (6)	0	7 (22)	1 (3)	1 (3)	0	0	0	1 (3)	0	0
Leukopenia	4 (13)	2 (6)	0	6 (19)	3 (9)	1 (3)	0	0	0	0	0	0
Neutropenia	8 (26)	4 (13)	1 (3)	6 (19)	3 (9)	3 (9)	0	0	0	0	0	0
Thrombocytopenia	10 (32)	2 (6)	4 (13)	6 (19)	1 (3)	2 (6)	0	0	0	0	0	0
												
*Other*												
Headache	12 (39)	0	0	12 (38)	3 (9)	0	24 (73)	1 (3)	0	16 (52)	0	0
Dyspnoea	7 (23)	0	1 (3)	5 (16)	1 (3)	0	1 (3)	1 (3)	0	3 (10)	0	0
Hypotension	6 (19)	0	0	1 (3)	0	0	0	0	0	0	0	0
Pain in extremity	1 (3)	0	0	5 (16)	1 (3)	0	0	0	0	1 (3)	0	0
Back pain	1 (3)	0	0	4 (13)	1 (3)	0	0	0	0	4 (13)	1 (3)	0
Cough	3 (10)	0	0	2 (6)	0	0	5 (15)	0	0	0	0	0
Uveitis	1 (3)	0	0	7 (22)	0	0	10 (30)	0	0	7 (23)	0	0

a Before crossover treatment.

Data presented as *n* (%). Individual adverse events presented here were reported by at least 10% of patients in any treatment arm.

**Table 3:** Efficacy

	**Dacarbazine+ placebo**	**Dacarbazine+10 mg kg^−1^ intetumumab**	**10 mg kg^−1^ Intetumumab**	**5 mg kg^−1^ Intetumumab**
Patients randomised	32	32	33	32
*PFS*	1.8 (0.0+, 13.4)	2.5 (0.0+, 14.6+)	1.4 (0.5, 18.6)	1.4 (0.0+, 5.8)
Hazard ratio (95% CI)		0.79 (0.46, 1.37)	1.25 (0.73, 2.14)	1.70 (0.99, 2.93)
*P*-value		0.47	0.36	0.07
				
*Event-free probability* a				
6-month PFS	14% (*n*=4)	17% (*n*=5)	19% (*n*=5)	0% (*n*=0)
				
*Objective tumour response*				
Patients evaluable	31	30	33	31
Tumour response (CR+PR)	3 (10)	1 (3)	2 (6)	0
PR	3 (10)	1 (3)	2 (6)	0
SD	10 (32)	16 (53)	8 (24)	8 (26)
PD	16 (52)	13 (43)	23 (70)	22 (71)
Disease control (SD or better)	13 (42)	17 (57)	10 (30)	8 (26)
Odds ratio (95% CI)		1.8 (0.7, 5.0)	0.6 (0.2, 1.7)	0.5 (0.2, 1.4)
*P*-value		0.35	0.31	0.17
				
*Overall survival*	8.0 (1.2, 29.0+)	11 (2.7, 26.4+)	15 (1.8, 33.8+)	9.8 (1.2, 35.4+)
Hazard ratio (95% CI)		0.78 (0.45, 1.33)	0.61 (0.35, 1.07)	0.97 (0.56, 1.68)
*P*-value		0.30	0.07	0.88
				
*Event-free probability* a				
1-year survival	34% (*n*=11)	47% (*n*=15)	64% (*n*=21)	26% (*n*=8)

Abbreviations: CI=confidence interval; CR=complete response; PD=progressive disease; PFS=progression-free survival; PR=partial response; SD=stable disease.

a *N*, number of patients at risk using Kaplan–Meier estimates.

Data presented as *n* (%) or median (range) unless noted otherwise. All durations are reported in months.
